# Opioid Dispensing Volume and Per-Patient Intensity in Sickle Cell Disease

**DOI:** 10.1001/jamanetworkopen.2026.32141

**Published:** 2026-09-01

**Authors:** Kevin Y. Xu, Brandon K. Attell, Joanna L. Buss, Tashalee R. Brown, Charles R. Jonassaint, Richard A. Grucza, Allison A. King, Max Jordan Nguemeni Tiako

**Affiliations:** Department of Psychiatry, Washington University School of Medicine, St Louis, Missouri; Center for Advancing Health Services, Policy & Economics Research, Washington University, St Louis, Missouri; Washington University School of Public Health, St Louis, Missouri; Georgia Health Policy Center, Georgia State University, Atlanta; Center for Advancing Health Services, Policy & Economics Research, Washington University, St Louis, Missouri; Department of Psychiatry, University of California Los Angeles David Geffen School of Medicine, Los Angeles; Department of Hematology and Medical Oncology, Emory University School of Medicine, Atlanta, Georgia; Department of Family and Community Medicine, Saint Louis University School of Medicine, St Louis, Missouri; Washington University School of Public Health, St Louis, Missouri; Department of Pediatrics, Division of Pediatric Hematology/Oncology, St Louis Children’s Hospital, Washington University School of Medicine, St Louis, Missouri; Department of Medicine, University of California Los Angeles David Geffen School of Medicine, Los Angeles

## Abstract

**IMPORTANCE:**

Opioids remain central to management of acute and chronic pain in sickle cell disease (SCD). Opioid dispensing declined over the 2010s amid opioid stewardship efforts, but patterns since 2020 are less clear. Analyses aggregating opioids into morphine milligram equivalents (MME) may obscure agent-specific patterns.

**OBJECTIVE:**

To characterize opioid dispensing volume and per-recipient frequency, duration, and dose among individuals with SCD.

**DESIGN, SETTING, AND PARTICIPANTS:**

A retrospective cohort study of commercially insured and Medicaid-enrolled patients with SCD using the Merative MarketScan databases across 3 periods from January 2011 to February 2016, March 2016 to December 2020, and January 2021 to December 2023.

**EXPOSURE:**

Calendar time across 3 prespecified periods: January 2011 to February 2016, March 2016 to December 2020, and January 2021 to December 2023.

**MAIN OUTCOMES AND MEASURES:**

Changes in opioid dispensing were examined across 3 periods (January 2011 to February 2016, March 2016 to December 2020, and January 2021 to December 2023). Segmented regression models estimated monthly percentage changes (MPCs) in 4 outcomes: (1) total dispensed prescriptions per 100 enrollees; and, among enrollees receiving opioids, (2) mean prescriptions per person, (3) mean days supplied, and (4) mean daily MMEs. Analyses included 6 opioids (hydromorphone, hydrocodone, morphine, tramadol, oxycodone, methadone) stratified by insurance type (commercial vs Medicaid).

**RESULTS:**

Across 48 760 individuals with SCD contributing person-time, 24 692 (50.6%) were Medicaid beneficiaries, 20 969 (43.0%) were aged 19 to 40 years, and 29 380 (60.3%) were female. From January 2021 through December 2023, total dispensing increased for all 6 agents among Medicaid beneficiaries, with the largest monthly increases for methadone (MPC, 1.76%; 95% CI, 1.24% to 2.29%) and hydromorphone (MPC, 1.43%; 95% CI, 1.00% to 1.86%). Among commercial enrollees, total dispensing increased for oxycodone (MPC, 0.84%; 95% CI, 0.60% to 1.07%) and morphine (MPC, 0.87%; 95% CI, 0.42% to 1.33%), was stable for hydrocodone, hydromorphone, and methadone, and declined for tramadol (MPC, −0.79%; 95% CI, −1.16% to −0.42%). Dispensed prescriptions increased only for oxycodone among commercially insured individuals (MPC, 0.14%; 95% CI, 0.08% to 0.20%) and tramadol among those with Medicaid (MPC, 0.96%; 95% CI, 0.56% to 1.36%). Mean days supplied and mean daily MMEs did not significantly increase for any opioid-insurance combination.

**CONCLUSIONS AND RELEVANCE:**

Among individuals with SCD, total opioid dispensing increased or stabilized during 2021 to 2023 after prior declines, most prominently among Medicaid beneficiaries, without broad increases in per-recipient frequency, duration, or dose. These patterns are consistent with broader distribution of opioid dispensing rather than treatment intensification among recipients.

## Introduction

Chronic pain is the leading contributor to poor quality of life among people living with sickle cell disease (SCD).^1–4^ Opioids are often necessary for managing acute and chronic pain episodes in SCD,^1,4,5^ and their use has been shaped by evolving state and national opioid stewardship policies as well as chronic pain clinical guidelines. Following the publication of the 2016 Centers for Disease Control and Prevention (CDC) Guideline for Prescribing Opioids for Chronic Pain^6^ and in response to the opioid overdose epidemic, US states increasingly implemented stricter opioid vigilance laws and prescription drug monitoring programs.^7,8^ While these policies, in some cases, led to appropriate reductions in high-risk prescribing,^7–10^ they also resulted in unintended reductions in opioid access among individuals with SCD and other populations with legitimate pain management needs, raising concerns about inadequate analgesia and increased pain-related hospitalizations.^11–15^ More recent analyses suggest opioid dispensing began to increase in some populations between 2020 and 2022,^11^ a period that also included COVID-19 disruption and recovery, evolving opioid prescribing policies and the release of updated guidelines emphasizing individualized, timely analgesia in SCD care by the American Society of Hematology.^16–18^

Despite these documented shifts in overall opioid dispensing patterns, much of the existing literature has examined opioid prescriptions in aggregate, potentially obscuring meaningful differences across individual opioid agents. Opioids differ substantially in potency, pharmacokinetics, regulatory classification, and clinical indication.^19^ For example, hydromorphone is approximately 5 times as potent as morphine; hydrocodone is commonly prescribed for short-term management of mild-to-moderate acute pain; and methadone is typically reserved for chronic pain management. In SCD care, these differences may correspond to different clinical roles and reflect the burden of chronic pain, pain-related comorbidities, and disease severity. Without agent-specific analyses that incorporate intensity (prescriptions per person), duration (days supplied), and dose (morphine milligram equivalents [MMEs]), observed changes in overall dispensing volume provide limited insight into how opioid use may be changing clinically. Few studies have examined agent-specific opioid dispensing, including prescriptions per person, days supplied, and daily MME, across the last decade. Accordingly, we examined dispensing of 6 commonly used opioid agents (hydromorphone, hydrocodone, morphine, tramadol, oxycodone, and methadone) among individuals with SCD from 2011 through 2023, characterizing total dispensing volume alongside per-recipient intensity, duration, and dose, separately by insurance type. This period encompassed 2 conceptually distinct categories of influence. The first was deliberate policy change: the 2016 and 2022 CDC opioid prescribing guidelines and the 2020 American Society of Hematology SCD pain guidelines, which may have altered dispensing through clinician behavior, institutional protocols, or patient access.^16^ The second was acute disruption from the COVID-19 pandemic, which may have affected dispensing through mechanisms largely independent of prescribing policy, including health care avoidance, capacity constraints, and shifts to telehealth.^17,18^ Because these influences overlapped in time, our analysis characterizes dispensing descriptively rather than attributing change to any single factor.

## Methods

### Study Design and Data Source

We analyzed data from the Merative MarketScan Commercial and Multi-State Medicaid Databases spanning January 1, 2011, through December 31, 2023. Models used 2 anchors, March 2016 and January 2021, to divide the study period into January 2011 to February 2016; March 2016 to December 2020; and January 2021 to December 2023. Data are deidentified and Health Insurance Portability and Accountability Act–compliant. The Washington University Human Research Protection Office deemed this study exempt, and informed consent was not required because the data were deidentified. The reporting of studies conducted using observational routinely collected health data statement for pharmacoepidemiology (RECORD-PE) guidelines for observational studies were followed.

### Study Population

Individuals with SCD were identified using validated *International Classification of Diseases, Ninth Revision (ICD-9)* and *International Statistical Classification of Diseases and Related Health Problems, Tenth Revision (ICD-10)* diagnosis codes (see eMethods in Supplement 1) if they had at least 1 inpatient encounter with an SCD diagnosis or at least 2 outpatient encounters with SCD diagnoses separated by at least 30 days during observable enrollment.^20–23^ We used serial monthly cross-sections of enrollees with SCD. Individuals contributed person-time to the monthly denominator while aged 1 to 64 years and enrolled, with outpatient pharmacy coverage, in a health plan contributing to the MarketScan databases. Monthly opioid dispensing outcomes were assigned according to the calendar month of the outpatient pharmacy fill. We stratified analyses by insurance type (commercial vs Medicaid). Continuous enrollment beyond that required for SCD ascertainment and monthly eligibility was not required, consistent with prior population-level time series.^11,12^ The derivation of the analytic sample is shown in eFigure 1 in Supplement 1.

### Exposure and Outcome Definitions

Outpatient opioids were identified using generic drug names and included 6 commonly dispensed agents: hydromorphone, hydrocodone, morphine, tramadol, oxycodone, and methadone. Our primary outcomes were (1) total prescriptions dispensed per 100 enrollees, and among individuals receiving opioids, (2) number of prescriptions per person, (3) mean days supplied, and (4) mean daily MMEs, calculated monthly for each opioid agent and insurance type. Recognizing that secular changes in the age distribution of the enrolled population may confound aggregate estimates,^24,25^ outcomes were age-standardized to the 2023 age distribution of SCD enrollees (see eMethods in Supplement 1).

### Statistical Analysis

For each opioid outcome, agent, and insurance group, we fitted segmented linear regression models^26^ with prespecified temporal anchors at March 2016 and January 2021 (see eMethods in Supplement 1). Models included a baseline monthly time trend, an immediate level-change term at each anchor, and a change-in-slope term at each anchor, allowing both level and slope to differ across January 2011 to February 2016; March 2016 to December 2020; and January 2021 to December 2023. Residual plots showed no persistent directional drift, and augmented Dickey-Fuller tests of model residuals rejected the unit-root null across all analyses, supporting residual stationarity. Monthly percentage changes (MPCs) were calculated as 100 × (exp(β) – 1), and 95% CIs were obtained by exponentiating the normal-approximation intervals of the log-scale segment-specific slope estimates (β ± 1.96 × SE). Data were analyzed from September 2025 to July 2026. All analyses were conducted using SAS version 9.4 (SAS Institute Inc). All tests were 2-sided, with statistical significance defined as α = .05.

## Results

The study cohort comprised 48 760 individuals aged 1 to 64 years with SCD who were enrolled in either commercial (24 068 [49.4%]) or Medicaid (24 692 [50.6%]) insurance at any time between January 1, 2011, and December 31, 2023 (eFigure 1 and eTable 1 in Supplement 1). Most individuals were female (29 380 [60.3%]). By age group, 18 590 (38.1%) were aged 1 to 18 years, 20 969 (43.0%) were aged 19 to 40 years, and 9201 (18.9%) were aged 41 to 64 years.

### Total Opioid Prescriptions Per 100 Enrollees

Oxycodone and hydrocodone were the most commonly dispensed opioids across the study period (Table). Among commercial enrollees, oxycodone averaged 5.60 (95% CI, 5.51–5.69) dispensed prescriptions per 100 enrollees per month and hydrocodone averaged 4.27 (95% CI, 4.04–4.50). Among Medicaid beneficiaries, the corresponding rates were 9.00 (95% CI, 8.72–9.27) and 4.59 (95% CI, 4.20–4.98), respectively.

Total dispensing declined across most opioids in both commercial and Medicaid-enrolled patients before 2021 (Figure 1 and Table). During January 2011 through February 2016, dispensing declined for 10 of 12 opioid-insurance combinations; the exceptions, both among commercial enrollees, were oxycodone (0.01% per month [0.1% per year]; 95% CI, −0.09% to 0.11%) and tramadol (0.23% per month [2.8% per year]; 95% CI, 0.07% to 0.40%). During March 2016 through December 2020, dispensing declined for all 6 opioids across both insurance groups.

During January 2021 through December 2023, the pre-2021 decline stabilized or reversed in most groups. Among Medicaid beneficiaries, all 6 agents increased significantly; the largest monthly increases were for methadone (1.76% per month [23.3% per year]; 95% CI, 1.24% to 2.29%) and hydromorphone (1.43% per month [18.6% per year]; 95% CI, 1.00% to 1.86%). Among commercial enrollees, oxycodone (0.84% per month [10.6% per year]; 95% CI, 0.60% to 1.07%) and morphine (0.87% per month [10.9% per year]; 95% CI, 0.42% to 1.33%) showed the greatest monthly increases. Tramadol increased modestly among Medicaid beneficiaries (0.58% per month [7.2% per year]; 95% CI, 0.09% to 1.08%) but declined among commercial enrollees (−0.79% per month [−9.1% per year]; 95% CI, −1.16% to −0.42%).

### Opioid Prescriptions Per Person

Among individuals receiving opioids, the number of dispensed prescriptions per person was close to 1 monthly prescription across most agents (Figure 2 and Table). In contrast to the increase in total dispensing volume, dispensed prescriptions per person did not increase broadly. During January 2011 through December 2020, prescriptions per person were stable or declining in nearly all groups, with no significant increase in any group (Table). During January 2021 through December 2023, they remained stable or declined for most opioids; the only significant increases were for oxycodone among commercial enrollees (0.14% per month [1.7% per year]; 95% CI, 0.08% to 0.20%) and tramadol among Medicaid beneficiaries (0.96% per month [12.1% per year]; 95% CI, 0.56% to 1.36%).

### Mean Days of Opioids Supplied

Mean days of opioids supplied per prescription varied by agent (Figure 3 and Table). Methadone had the longest duration, averaging 47.18 days (95% CI, 44.42–49.94 days) among commercial enrollees and 42.59 days (95% CI, 40.81– 44.37 days) among Medicaid beneficiaries. Morphine averaged 37.59 days (95% CI, 35.83–39.34 days) and 32.85 days (95% CI, 31.92–33.79 days), respectively. Hydrocodone had the shortest duration at 14.39 days (95% CI, 14.05–14.72 days) and 12.20 days (95% CI, 11.93–12.48 days).

Mean days supplied were generally stable or declining for most opioids before 2021 (Table). During January 2021 through December 2023, every opioid in both insurance groups was stable or declining, with no significant increase in any group. The steepest decline was for methadone among commercial enrollees (−2.72% per month [−28.2% per year]; 95% CI, −3.71% to −1.71%).

### Mean Daily MME

Mean daily opioid dose, measured in MME, varied by agent. Methadone had the highest mean daily MME at 175.28 (95% CI, 165.48–185.07) among commercial enrollees and 170.08 (95% CI, 164.71–175.45) among Medicaid beneficiaries, followed by hydromorphone (commercial: 130.36; 95% CI, 127.14–133.57; Medicaid: 108.57; 95% CI, 105.53–111.61). Tramadol had the lowest mean daily MME (commercial: 38.61; 95% CI, 37.64–39.59; Medicaid: 35.57; 95% CI, 34.37–36.77). During March 2016 through December 2020, mean daily MME declined for every opioid in both insurance groups (Figure 4 and Table).

During January 2021 through December 2023, no opioid in either insurance group showed a significant increase in mean daily MME. Morphine declined in both groups (commercial, −0.51% per month [−6.0% per year]; 95% CI, −0.97% to −0.05%; Medicaid, −0.64% per month [−7.4% per year]; 95% CI, −1.12% to −0.16%), and hydrocodone declined among Medicaid beneficiaries (−0.72% per month [−8.3% per year]; 95% CI, −0.93% to −0.50%); no other agent changed significantly in terms of mean daily MME.

## Discussion

This cohort study found that total opioid dispensing per 100 enrollees largely declined before 2021, with declines observed across all opioid agents and insurance groups during the 2016 to 2020 period. During 2021 to 2023, total opioid dispensing per 100 enrollees stabilized or increased for most opioids in both insurance groups, particularly among Medicaid beneficiaries, but this was not accompanied by broad increases in per-recipient dispensing intensity. Dispensed prescriptions per person, mean days supplied, and mean daily MME generally remained stable or declined.

The decline in dispensing up to 2021 may reflect multiple factors, including national opioid stewardship efforts, payer policies, and broader changes in pain management. During 2021 to 2023, total opioid dispensing per 100 enrollees stabilized or increased for most opioids in both insurance groups, but dispensed prescriptions per person, mean days supplied, and mean daily MME did not show corresponding increases. This pattern may reflect wider distribution of prescriptions across patients rather than intensification of opioid therapy among individuals already receiving opioids. Among Medicaid beneficiaries, for example, hydromorphone rose in total dispensing yet showed fewer prescriptions per person and shorter days supplied (annualizing the monthly rates, roughly −7% and −14% per year), consistent with wider distribution rather than more intensive use.

The observed differences in opioid dispensing trends between Medicaid and commercially insured populations may reflect differences in policy environments, patient characteristics, access to SCD care, and clinical decision-making. Prior to 2021, total opioid dispensing declined more steeply among Medicaid beneficiaries than among commercially insured individuals; this was most pronounced for high-potency agents (eg, hydromorphone), mirroring declines documented in the general population.^9^ While the mechanisms cannot be established from claims data alone, the timing of these changes overlaps with the expansion of prescription drug monitoring programs and utilization management policies.^27^ Medicaid beneficiaries may be more directly affected by state-level prescribing restrictions, prior authorization requirements, and other payer policies, which could differentially impact access to opioid therapy for patients with SCD.^28–30^ These declines gave way to stabilization or modest increases in total dispensing for most opioids in both groups, without parallel increases in prescriptions per person, days supplied, or mean daily MME, again consistent with broader distribution across patients rather than intensification.

Notably, among Medicaid beneficiaries, tramadol rose in both total dispensing and prescriptions per person (by approximately 7% and 12% per year), the only agent for which per-recipient use increased alongside volume. Its Schedule IV classification and lower potency may make it more acceptable to clinicians or payers than Schedule II opioids, which may explain why its use rose as dispensing of more potent agents stabilized or fell.^31^ In the SCD context, lower potency opioids such as tramadol may provide inadequate analgesia for some patients, potentially leading to repeated dispensing without adequate pain control.^31^ Tramadol also has specific safety concerns, including complex metabolism, drug-drug interactions, and accumulation of active metabolites, particularly in the setting of kidney impairment, a known complication of SCD.^32^

### Limitations and Strengths

This study had limitations. First, we could not identify enrollees’ state of residence in MarketScan data, so we could not adjust or stratify by state. This matters in SCD, where care delivery, access to specialized treatment, opioid-prescribing policy, and even survival differ substantially across states. Compounding this, the database’s composition changes over time as insurers enter or leave, shifting the geographic mix of the sample. Because opioid dispensing varies regionally, both unmeasured state-level differences and these changes in geographic composition may have contributed to observed temporal trends. Second, although our use of age standardization accounts for shifts in population structure over time, residual confounding from unmeasured factors remains possible. Because the study period included overlapping clinical and policy changes, the analysis cannot separate the effects of the 2016 CDC guideline, the 2020 American Society of Hematology guideline, COVID-19–related disruption and recovery, and other contemporaneous changes in opioid policy and health care delivery.^17,18^

Despite these limitations, our study is strengthened by a large, multistate sample of nearly 50 000 individuals with SCD, one of the largest administrative cohorts examining opioid dispensing to people living with SCD in the US to our knowledge. The use of pharmacy claims data allows for comprehensive capture of outpatient prescription fills, and our agent-specific approach with multiple dispensing dimensions provides granular insight into opioid exposure patterns.

## Conclusions

In this cohort study, a national analysis of individuals with SCD, opioid dispensing generally declined before January 2021, followed by stabilization or increases in total dispensing volume during the 2021 to 2023 period, particularly among Medicaid beneficiaries. These increases were not accompanied by broad increases in dispensed opioid prescriptions per person, days supplied, or mean daily MME, suggesting that changes in total dispensing volume may reflect broader distribution of prescriptions rather than intensification of opioid therapy among individuals already receiving opioids. As the treatment landscape for SCD continues to evolve, including the emergence of gene therapies and other curative approaches,^33^ ensuring equitable access to the full spectrum of disease-modifying and symptom management strategies will be essential.

## Supplementary Material

Online Only Supplement 1 SUPPLEMENT 1.
eTable. Enrollment Characteristics of Individuals With Sickle Cell Disease Included in the Study (n=48,760)
eFigure. Derivation of the Analytic Sample
eMethods. Variables and Administrative Codes

Online Only Supplement 2 SUPPLEMENT 2.
Data Sharing Statement

## Figures and Tables

**Figure 1.**
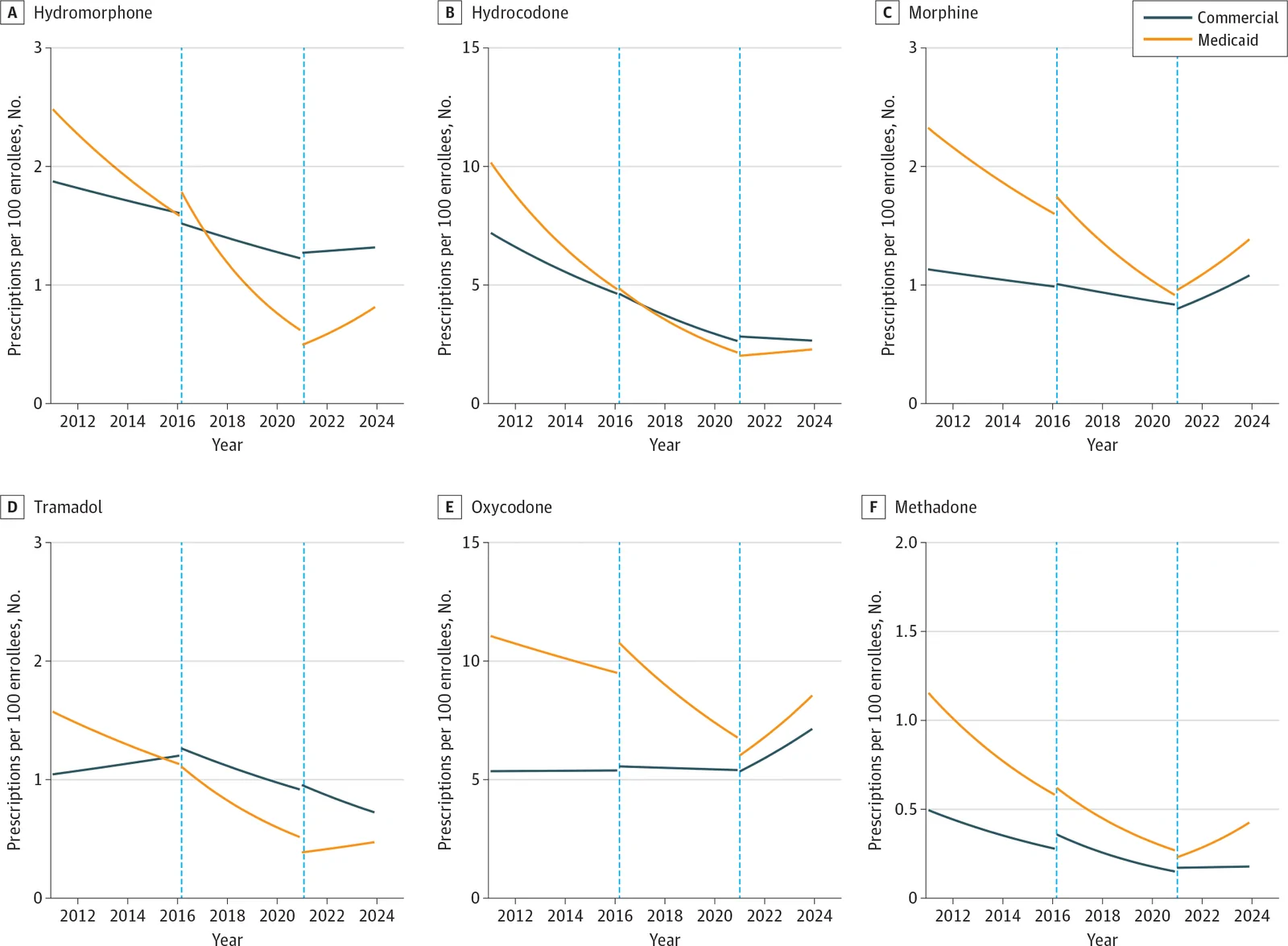
Line Charts of Monthly Dispensed Opioid Prescriptions Per 100 Enrollees for 6 Opioid Analgesics by Insurance Type, 2011 to 2023 Lines are fitted values from segmented regression models estimated on the logarithmic scale and displayed on the natural scale. Dashed vertical lines mark the dates of March 2016 and January 2021, when models allowed both an immediate level change and a change in slope. Vertical discontinuities therefore reflect modeled level changes rather than gaps in the underlying data.

**Figure 2.**
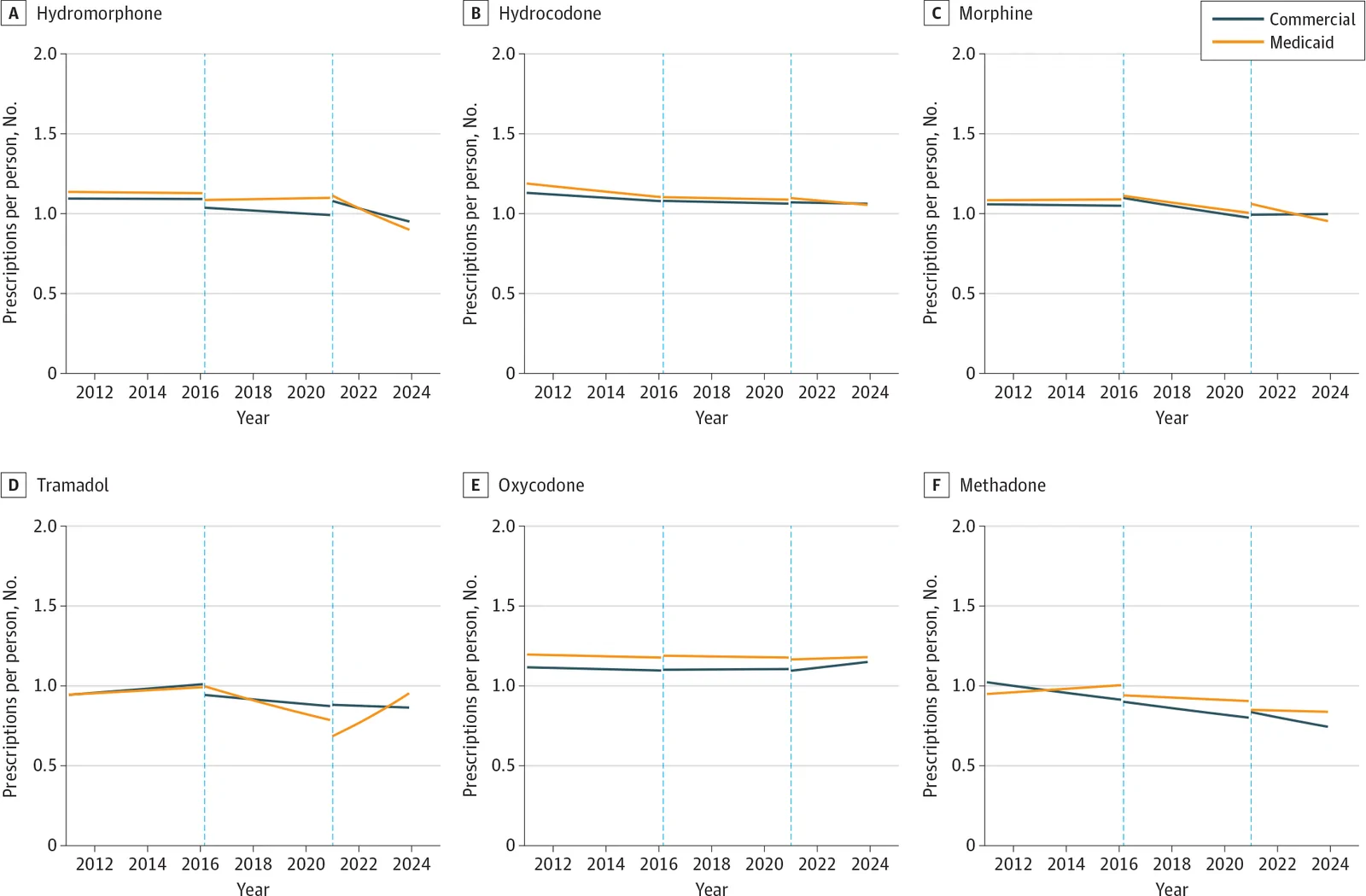
Line Charts of Monthly Mean Number of Dispensed Prescriptions Per Person Among Opioid Recipients by Insurance Type, 2011 to 2023 Fitted values were estimated using segmented regression models on the logarithmic scale and plotted on the natural scale. The dashed vertical lines denote March 2016 and January 2021, at which immediate level changes and slope changes were permitted; breaks in fitted lines represent modeled level changes, not missing data.

**Figure 3.**
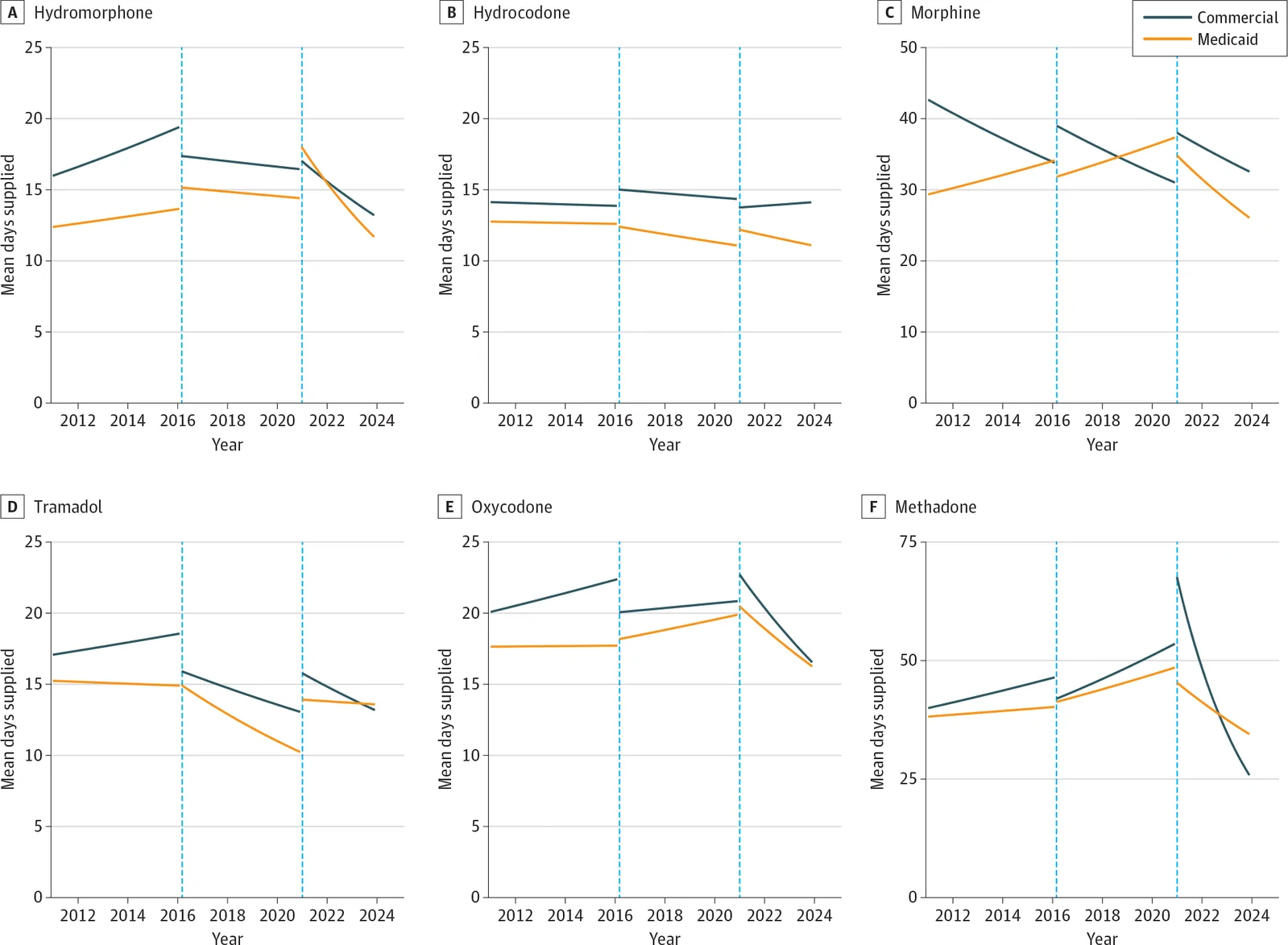
Line Charts of Monthly Mean Days Supplied Per Dispensed Opioid Prescription by Insurance Type, 2011 to 2023 In March 2016 and January 2021, models allowed an immediate change in level as well as a change in slope. Discontinuities at these dashed vertical lines are therefore model-estimated level changes rather than interruptions in observed data.

**Figure 4.**
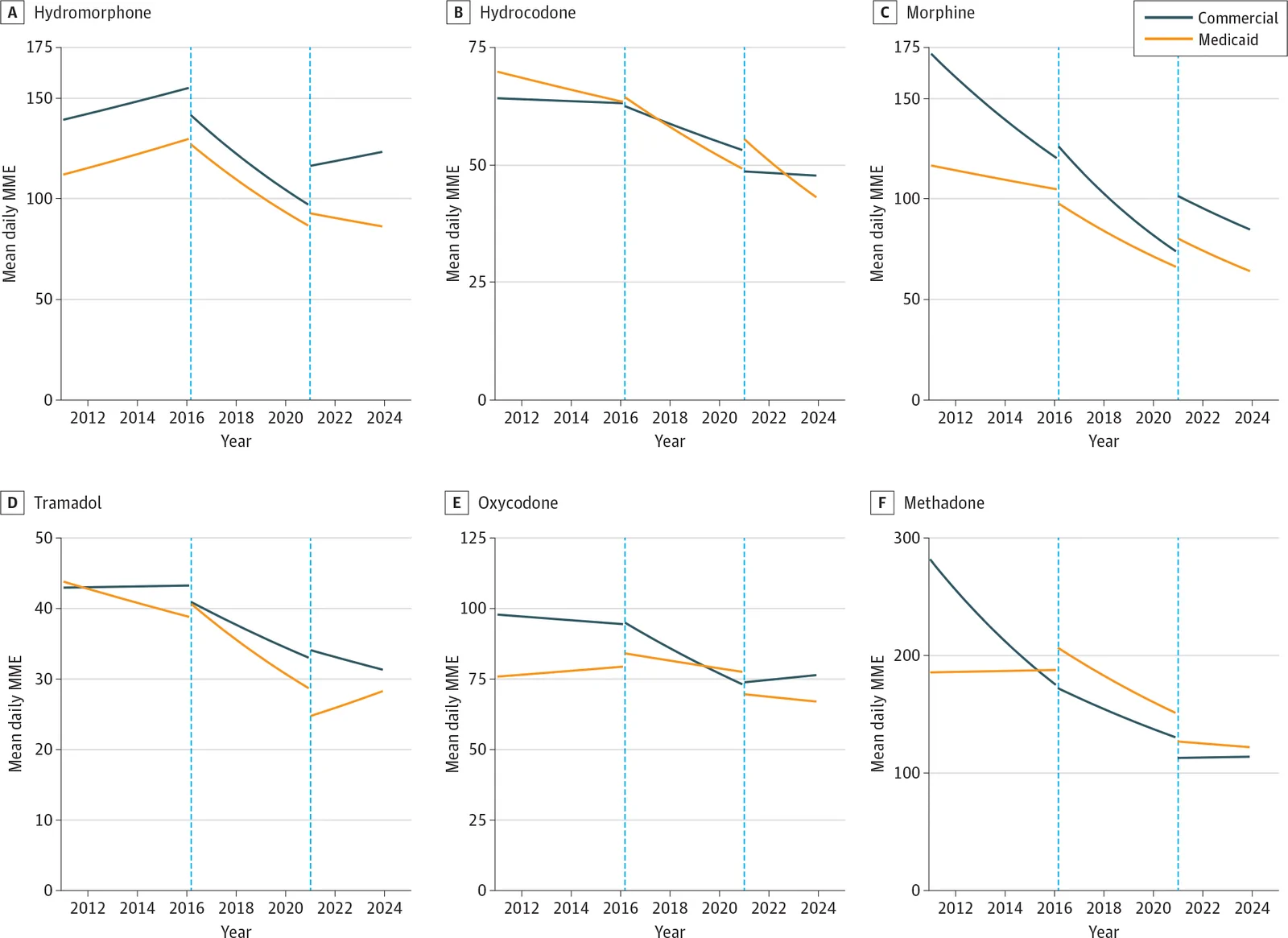
Line Charts of Monthly Mean Daily Morphine Milligram Equivalents (MMEs) Among Opioid Recipients by Insurance Type, 2011 to 2023 Segmented regression models were estimated on the logarithmic scale, with fitted values displayed on the natural scale. Dashed vertical lines denote March 2016 and January 2021, at which models allowed both an immediate level change and a change in slope; discontinuities at these dates reflect modeled level changes rather than gaps in the underlying data.

**Table.** Opioid Dispensing Trends Among Individuals With Sickle Cell Disease, 2011 to 2023

Opioid and insurance	Mean (95% CI)^a^	MPC (95% CI)		
		January 2011 to February 2016	March 2016 to December 2020	January 2021 to December 2023
**Total dispensed prescriptions per 100 people per mo**				
Hydrocodone				
Commercial	4.27 (4.04 to 4.50)	−0.72 (−0.84 to −0.60)	−0.99 (−1.12 to −0.85)	−0.18 (−0.46 to 0.10)
Medicaid	4.59 (4.20 to 4.98)	−1.22 (−1.34 to −1.10)	−1.42 (−1.56 to −1.29)	0.36 (0.08 to 0.64)
Hydromorphone				
Commercial	1.51 (1.47 to 1.55)	−0.25 (−0.39 to −0.11)	−0.37 (−0.53 to −0.22)	0.10 (−0.22 to 0.42)
Medicaid	1.37 (1.26 to 1.47)	−0.73 (−0.92 to −0.55)	−1.83 (−2.04 to −1.63)	1.43 (1.00 to 1.86)
Methadone				
Commercial	0.28 (0.27 to 0.30)	−0.94 (−1.29 to −0.58)	−1.50 (−1.88 to −1.12)	0.11 (−0.67 to 0.91)
Medicaid	0.57 (0.53 to 0.62)	−1.12 (−1.34 to −0.89)	−1.45 (−1.70 to −1.20)	1.76 (1.24 to 2.29)
Morphine				
Commercial	0.98 (0.96 to 1.01)	−0.23 (−0.43 to −0.02)	−0.33 (−0.55 to −0.11)	0.87 (0.42 to 1.33)
Medicaid	1.53 (1.45 to 1.60)	−0.61 (−0.77 to −0.45)	−1.13 (−1.31 to −0.95)	1.07 (0.70 to 1.44)
Oxycodone				
Commercial	5.60 (5.51 to 5.69)	0.01 (−0.09 to 0.11)	−0.05 (−0.16 to 0.06)	0.84 (0.60 to 1.07)
Medicaid	9.00 (8.72 to 9.27)	−0.25 (−0.38 to −0.11)	−0.81 (−0.96 to −0.66)	1.01 (0.70 to 1.32)
Tramadol				
Commercial	1.05 (1.02 to 1.07)	0.23 (0.07 to 0.40)	−0.56 (−0.74 to −0.38)	−0.79 (−1.16 to −0.42)
Medicaid	0.93 (0.86 to 0.99)	−0.54 (−0.76 to −0.33)	−1.34 (−1.58 to −1.11)	0.58 (0.09 to 1.08)
**Dispensed prescriptions per person**				
Hydrocodone				
Commercial	1.08 (1.08 to 1.09)	−0.08 (−0.11 to −0.05%)	−0.03 (−0.06 to 0.00)	−0.02 (−0.09 to 0.04)
Medicaid	1.11 (1.10 to 1.12)	−0.12 (−0.15 to −0.09)	−0.02 (−0.05 to 0.01)	−0.11 (−0.17 to −0.05)
Hydromorphone				
Commercial	1.05 (1.03 to 1.06)	0.00 (−0.11 to 0.10)	−0.08 (−0.19 to 0.03)	−0.36 (−0.59 to −0.14)
Medicaid	1.09 (1.07 to 1.12)	−0.01 (−0.17 to 0.14)	0.02 (−0.15 to 0.19)	−0.61 (−0.96 to −0.26)
Methadone				
Commercial	0.89 (0.87 to 0.91)	−0.19 (−0.36 to −0.01)	−0.21 (−0.40 to −0.01)	−0.34 (−0.73 to 0.06)
Medicaid	0.93 (0.91 to 0.95)	0.09 (−0.07 to 0.26)	−0.07 (−0.25 to 0.11)	−0.04 (−0.41 to 0.33)
Morphine				
Commercial	1.03 (1.02 to 1.04)	−0.01 (−0.09 to 0.06)	−0.21 (−0.29 to −0.13)	0.01 (−0.15 to 0.18)
Medicaid	1.06 (1.05 to 1.08)	0.01 (−0.08 to 0.10)	−0.18 (−0.28 to −0.08)	−0.31 (−0.55 to −0.07)
Oxycodone				
Commercial	1.11 (1.10 to 1.11)	−0.03 (−0.06 to 0.00)	0.01 (−0.02 to 0.04)	0.14 (0.08 to 0.20)
Medicaid	1.18 (1.18 to 1.19)	−0.03 (−0.05 to 0.00)	−0.02 (−0.05 to 0.01)	0.04 (−0.02 to 0.09)
Tramadol				
Commercial	0.93 (0.92 to 0.95)	0.11 (−0.01 to 0.23)	−0.13 (−0.27 to 0.00%)	−0.06 (−0.33 to 0.22%)
Medicaid	0.91 (0.89 to 0.93)	0.08 (−0.09 to 0.25)	−0.42 (−0.61 to −0.23)	0.96 (0.56 to 1.36)
**Mean days supplied**				
Hydrocodone				
Commercial	14.39 (14.05 to 14.72)	−0.03 (−0.23 to 0.17)	−0.08 (−0.30 to 0.14)	0.08 (−0.37 to 0.52)
Medicaid	12.20 (11.93 to 12.48)	−0.02 (−0.21 to 0.17)	−0.20 (−0.41 to 0.01)	−0.27 (−0.70 to 0.16)
Hydromorphone				
Commercial	17.19 (16.50 to 17.89)	0.32 (0.00 to 0.64)	−0.10 (−0.45 to 0.26)	−0.72 (−1.44 to 0.00)
Medicaid	14.25 (13.80 to 14.70)	0.16 (−0.08 to 0.40)	−0.09 (−0.35 to 0.18)	−1.22 (−1.76 to −0.69)
Methadone				
Commercial	47.18 (44.42 to 49.94)	0.25 (−0.21 to 0.70)	0.43 (−0.08 to 0.94)	−2.72 (−3.71 to −1.71)
Medicaid	42.59 (40.81 to 44.37)	0.09 (−0.25 to 0.42)	0.28 (−0.09 to 0.65)	−0.78 (−1.53 to −0.03)
Morphine				
Commercial	37.59 (35.83 to 39.34)	−0.38 (−0.76 to 0.00)	−0.40 (−0.82 to 0.02)	−0.44 (−1.30 to 0.42)
Medicaid	32.85 (31.92 to 33.79)	0.25 (0.01 to 0.49)	0.28 (0.02 to 0.54)	−0.83 (−1.35 to −0.31)
Oxycodone				
Commercial	20.75 (20.23 to 21.28)	0.18 (−0.03 to 0.39)	0.07 (−0.16 to 0.29)	−0.90 (−1.35 to −0.44)
Medicaid	18.46 (18.09 to 18.84)	0.01 (−0.16 to 0.17)	0.16 (−0.02 to 0.34)	−0.65 (−1.02 to −0.29)
Tramadol				
Commercial	16.15 (15.51 to 16.79)	0.14 (−0.18 to 0.45%)	−0.35 (−0.69 to 0.00)	−0.51 (−1.20 to 0.18)
Medicaid	14.13 (13.57 to 14.68)	−0.04 (−0.35 to 0.28)	−0.66 (−1.00 to −0.32)	−0.07 (−0.77 to 0.64)
**Mean daily MME**				
Hydrocodone				
Commercial	58.15 (56.90 to 59.40)	−0.03 (−0.15 to 0.10%)	−0.28 (−0.42 to −0.15)	−0.05 (−0.33 to 0.23)
Medicaid	58.97 (57.60 to 60.33)	−0.16 (−0.25 to −0.06)	−0.47 (−0.58 to −0.36)	−0.72 (−0.93 to −0.50)
Hydromorphone				
Commercial	130.36 (127.14 to 133.57)	0.18 (0.04 to 0.31)	−0.65 (−0.80 to −0.51)	0.16 (−0.14 to 0.47)
Medicaid	108.57 (105.53 to 111.61)	0.24 (0.08 to 0.40)	−0.66 (−0.84 to −0.49)	−0.21 (−0.57 to 0.16)
Methadone				
Commercial	175.28 (165.48 to 185.07)	−0.78 (−1.11 to −0.44)	−0.49 (−0.86 to −0.11%)	0.03 (−0.74 to 0.79)
Medicaid	170.08 (164.71 to 175.45)	0.02 (−0.16 to 0.20)	−0.55 (−0.74 to −0.35)	−0.11 (−0.52 to 0.29)
Morphine				
Commercial	116.20 (110.92 to 121.48)	−0.59 (−0.79 to −0.38)	−0.93 (−1.15 to −0.71)	−0.51 (−0.97 to −0.05)
Medicaid	91.62 (88.20 to 95.04)	−0.17 (−0.39 to 0.04)	−0.68 (−0.91 to −0.44)	−0.64 (−1.12 to −0.16)
Oxycodone				
Commercial	87.03 (85.11 to 88.95)	−0.06 (−0.18 to 0.06)	−0.45 (−0.58 to −0.32)	0.10 (−0.17 to 0.37)
Medicaid	76.86 (75.93 to 77.79)	0.07 (0.02 to 0.13)	−0.14 (−0.20 to −0.08)	−0.11 (−0.24 to 0.02)
Tramadol				
Commercial	38.61 (37.64 to 39.59)	0.01 (−0.14 to 0.17)	−0.38 (−0.54 to −0.21)	−0.24 (−0.58 to 0.10)
Medicaid	35.57 (34.37 to 36.77)	−0.20 (−0.37 to −0.02)	−0.61 (−0.80 to −0.42)	0.38 (−0.02 to 0.78)

Abbreviations: MME, morphine milligram equivalents; MPC, monthly percentage changes.

a The mean (95% CI) column is the mean monthly value of each outcome over the full study period (January 2011 to December 2023).

## Data Availability

See Supplement 2.

## Key Points

### Question

Did opioid dispensing volume and per-recipient frequency, duration, and dose change among people with sickle cell disease amid national opioid stewardship responses to the overdose crisis?

### Findings

In this cohort study of 48 760 individuals with sickle cell disease, monthly slopes for total opioid dispensing were generally negative across agents before 2021; dispensing stabilized or increased for most agents during the 2021 to 2023 period, particularly among Medicaid beneficiaries. Per-recipient frequency, duration, and dose did not broadly increase.

### Meaning

These findings suggest recent increases in total opioid dispensing among individuals with sickle cell disease are consistent with broader distribution of prescriptions rather than intensification of regimens among existing recipients.

## References

[1] Osunkwo I, O’Connor HF, Saah E. Optimizing the management of chronic pain in sickle cell disease. Hematology Am Soc Hematol Educ Program. 2020;2020(1):562–569. doi: 10.1182/hematology.2020000143 PMC7727591 33275672

[2] Ballas SK, Gupta K, Adams-Graves P. Sickle cell pain: a critical reappraisal. Blood. 2012;120(18):3647–3656. doi:10.1182/blood-2012-04-383430 22923496

[3] Ford CL, Airhihenbuwa CO. The public health critical race methodology: praxis for antiracism research. Soc Sci Med. 2010;71(8):1390–1398. doi:10.1016/j.socscimed.2010.07.030 20822840

[4] Ballas SK. Sickle cell disease: Classification of clinical complications and approaches to preventive and therapeutic management. Clin Hemorheol Microcirc. 2018;68(2–3):105–128. doi:10.3233/CH-189002 29614627

[5] Carroll CP. Opioid treatment for acute and chronic pain in patients with sickle cell disease. Neurosci Lett. 2020;714:134534. doi:10.1016/j.neulet.2019.134534 31593753

[6] Dowell D, Haegerich TM, Chou R. CDC guideline for prescribing opioids for chronic pain–United States, 2016. JAMA. 2016;315(15):1624–1645. doi:10.1001/jama.2016.1464 PMC6390846 26977696

[7] Puac-Polanco V, Chihuri S, Fink DS, Cerdá M, Keyes KM, Li G. Prescription drug monitoring programs and prescription opioid-related outcomes in the United States. Epidemiol Rev. 2020;42(1):134–153. doi:10.1093/epirev/mxaa002 PMC7947593 32242239

[8] McGinty EE, Bicket MC, Seewald NJ, Effects of state opioid prescribing laws on use of opioid and other pain treatments among commercially insured U.S. adults. Ann Intern Med. 2022;175(5):617–627. doi:10.7326/M21-4363 PMC9277518 35286141

[9] Bohnert ASB, Guy GP Jr, Losby JL. Opioid prescribing in the United States before and after the centers for disease control and prevention’s 2016 opioid guideline. Ann Intern Med. 2018;169(6):367–375. doi:10.7326/M18-1243 PMC6176709 30167651

[10] Han J, Zhou J, Saraf SL, Gordeuk VR, Calip GS. Characterization of opioid use in sickle cell disease. Pharmacoepidemiol Drug Saf. 2018;27(5):479–486. doi:10.1002/pds.4291 PMC5815963 28815799

[11] Xu KY, Newman TV, McGill LS, Ten-year trends in opioid prescribing and vaso-occlusive crises in sickle cell disease: a population-based national cohort study (2011–2022). Lancet Reg Health Am. 2025;50:101214. doi:10.1016/j.lana.2025.101214 PMC12396581 40894271

[12] Kang HA, Wang B, Barner JC, Opioid prescribing and outcomes in patients with sickle cell disease post-2016 CDC guideline. JAMA Intern Med. 2024;184(5):510–518. doi:10.1001/jamainternmed.2023.8538 PMC10928539 38466269

[13] Liu SA, Brown TR, King AA, Opioid-related emergency admissions in people with opioid dependence/use disorder with and without sickle cell disease: an analysis of multi-state insurance claims. Gen Hosp Psychiatry. 2024;91:83–88. doi:10.1016/j.genhosppsych.2024.09.013 PMC11634658 39378616

[14] Brown TR, Jansen MO, Rolin SA, Liu SA, Xu KY. The misuse of malingering diagnoses in individuals with sickle cell disease. Gen Hosp Psychiatry. 2024;87:157–158. doi:10.1016/j.genhosppsych.2023.12.001 PMC10982992 38102021

[15] Ruta NS, Ballas SK. The opioid drug epidemic and sickle cell disease: guilt by association. Pain Med. 2016;17(10):1793–1798. doi:10.1093/pm/pnw074 27152018

[16] Brandow AM, Carroll CP, Creary S, American Society of Hematology 2020 guidelines for sickle cell disease: management of acute and chronic pain. Blood Adv. 2020;4(12):2656–2701. doi:10.1182/bloodadvances.2020001851 PMC7322963 32559294

[17] Reeves SL, Plegue M, Patel PN, Assessing patterns of telehealth use among people with sickle cell disease enrolled in medicaid during the start of the COVID-19 pandemic. Telemed J E Health. 2024;30(7):e1971–e1979. doi:10.1089/tmj.2023.0422 PMC11257828 38603584

[18] Attell BK, Plaxco AP, Zhou M, Emergency department utilization before and during the COVID-19 pandemic among individuals with sickle cell disease. BMC Emerg Med. 2024;24(1):134. doi:10.1186/s12873-024-01043-5 PMC11287848 39075379

[19] Adams MCB, Sward KA, Perkins ML, Hurley RW. Standardizing research methods for opioid dose comparison: the NIH HEAL morphine milligram equivalent calculator. Pain. 2025;166(8):1729–1737. doi:10.1097/j.pain.0000000000003529 PMC12266977 39907478

[20] Grosse SD, Green NS, Reeves SL. Administrative data identify sickle cell disease: a critical review of approaches in U.S. health services research. Pediatr Blood Cancer. 2020;67(12):e28703. doi:10.1002/pbc.28703 PMC7606824 32939942

[21] Reeves SL, Madden B, Wu M, Performance of ICD-10-CM diagnosis codes for identifying children with sickle cell anemia. Health Serv Res. 2020;55(2):310–317. doi:10.1111/1475-6773.13257 PMC7080376 31916247

[22] Cronin RM, Lin CJ, Chiang C, MacEwan SR, DeBaun MR, Hyer JM. The use of FDA-approved medications for preventing vaso-occlusive events in sickle cell disease. Blood Adv. 2023;7(13):3114–3116. doi:10.1182/bloodadvances.2022008965 PMC10362533 36883847

[23] Newman TV, Yang J, Suh K, Jonassaint CR, Kane-Gill SL, Novelli EM. Use of disease-modifying treatments in patients with sickle cell disease. JAMA Netw Open. 2023;6(11):e2344546. doi:10.1001/jamanetworkopen.2023.44546 PMC10665975 37991760

[24] Snyder AB, Zhou M, Attell BK, Opioid use after first opioid prescription in children with sickle cell disease. JAMA Pediatr. 2024;178(4):408–410. doi:10.1001/jamapediatrics.2023.6500 PMC10877502 38372994

[25] DeBaun MR, Ghafuri DL, Rodeghier M, Decreased median survival of adults with sickle cell disease after adjusting for left truncation bias: a pooled analysis. Blood. 2019;133(6):615–617. doi:10.1182/blood-2018-10-880575 PMC6367648 30530750

[26] Wagner AK, Soumerai SB, Zhang F, Ross-Degnan D. Segmented regression analysis of interrupted time series studies in medication use research. J Clin Pharm Ther. 2002;27(4):299–309. doi:10.1046/j.1365-2710.2002.00430.x 12174032

[27] Jayawardhana J, Abraham AJ, Young HN, Perri M III. Opioids in Georgia medicaid: gender and insurance disparities in utilization and potential inappropriate prescribing practices. J Pharm Health Serv Res. 2018;9(2):101–108. doi:10.1111/jphs.12215 PMC6051715 30034551

[28] Zhang H, Kilaru AS, Meisel ZF, Bao Y. Prescription drug monitoring program mandates and opioids dispensed following emergency department encounters for patients with sickle cell disease or cancer with bone metastasis. JAMA. 2021;326(3):274–276. doi:10.1001/jama.2021.10161 PMC8204257 34125882

[29] Sinha CB, Bakshi N, Ross D, Krishnamurti L. Management of chronic pain in adults living with sickle cell disease in the era of the opioid epidemic: a qualitative study. JAMA Netw Open. 2019;2(5):e194410. doi:10.1001/jamanetworkopen.2019.4410 PMC6632133 31125105

[30] McGee BT, Attell BK, Marton J, Changes in acute care use among people with sickle cell disease after adoption of a prescription drug monitoring program. Blood Adv. 2025;9(12):3014–3023. doi:10.1182/bloodadvances.2024013594 PMC12205578 39993232

[31] Dunn KE, Bergeria CL, Huhn AS, Strain EC. A systematic review of laboratory evidence for the abuse potential of tramadol in humans. Front Psychiatry. 2019;10:704. doi:10.3389/fpsyt.2019.00704 PMC6775208 31616329

[32] Valle J, Lebensburger JD, Garimella PS, Gopal S. Prevalence, mortality, and access to care for chronic kidney disease in medicaid-enrolled adults with sickle cell disease in California: retrospective cohort study. JMIR Public Health Surveill. 2024;10:e57290. doi:10.2196/57290 PMC11287091 39008353

[33] Cavazzana M, Corsia A, Brusson M, Miccio A, Semeraro M. Treating sickle cell disease: gene therapy approaches. Annu Rev Pharmacol Toxicol. 2025;65(1):397–413. doi:10.1146/annurev-pharmtox-022124-022000 39259977

