# Supplementary material for: Opioid Dispensing Volume and Per-Patient Intensity in Sickle Cell Disease
Source: JAMA Netw Open. Author manuscript; Available in PMC 2026 Oct 1. (PMC13629608; doi:10.1001/jamanetworkopen.2026.32141)
Supplement: Supplementary file 1 [file NIHMS2208928-supplement-Online_Only_Supplement_1.pdf]

Supplemental Online Content

Xu KY, Attell BK, Buss JL, et al. Opioid dispensing volume and per-patient intensity in sickle cell disease. *JAMA Netw Open.* 2026;9(9):e2632141. doi:10.1001/jamanetworkopen.2026.32141

eTable 1 : Enrollment characteristics of individuals with sickle cell disease included in the study (n=48,760)

eFigure 1. Derivation of the Analytic Sample

eMethods. Variables and Administrative Codes

This supplemental material has been provided by the authors to give readers additional information about their work.

© 2026 Xu KY et al. *JAMA Network Open*.

eTable 1: Enrollment characteristics of individuals with sickle cell disease included in the study (n=48,760)

	N	%
Insurance Type		
Commercial	24068	49.4
Medicaid	24692	50.6
Sex		
Male	19380	39.8
Female	29380	60.2
Age, years		
1-18	18590	38.1
19-40	20969	43.0
41-64	9201	18.9
Race/Ethnicity (Medicaid enrollees only, n = 24,692)		
Hispanic	329	1.3
Non-Hispanic Black	18644	75.5
Non-Hispanic White	938	3.8
Other	3508	14.2
Missing	1273	5.2
Region of US (Commercial enrollees only, n = 24,068)		
South	14413	59.9
Northeast	4176	17.3
North Central	3447	14.3
West	1698	7.1
Unknown	334	1.4
Enrollment Year		
2011	12987	26.6
2012-2015	16172	33.2
2016-2019	11824	24.3
2020-2023	7777	15.9

© 2026 Xu KY et al. *JAMA Network Open*.

eFigure 1. Derivation of the Analytic Sample

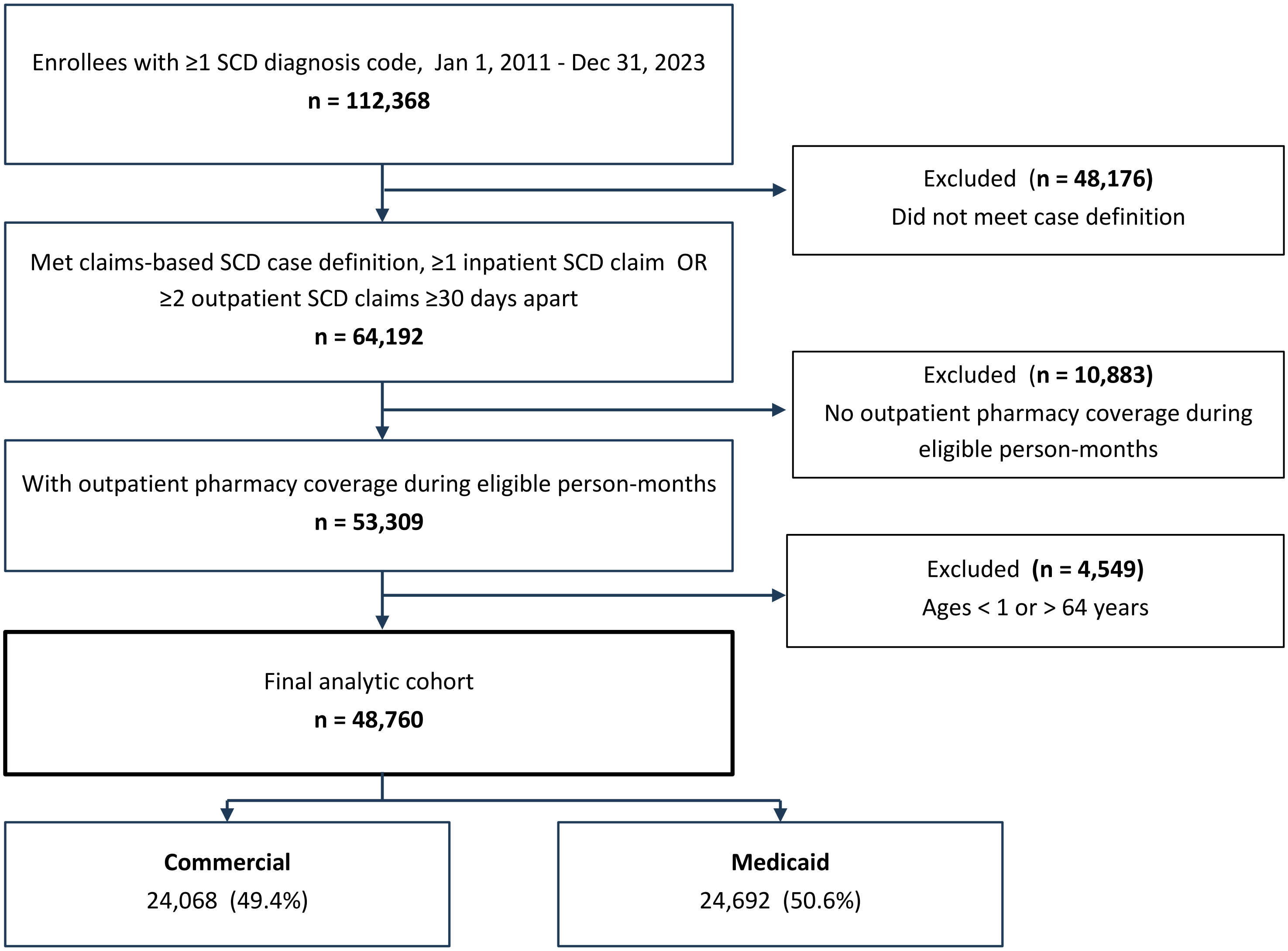

© 2026 Xu KY et al. *JAMA Network Open*.

eMethods. Variables and Administrative Codes

Variable	Administrative Codes
Diagnosis of Sickle Cell Disease (SCD)	Individuals with SCD were identified using a validated claims-based algorithm applied across the full duration of insurance enrollment. Eligibility required either at least one inpatient claim with an SCD diagnosis or at least two outpatient claims with an SCD diagnosis separated by ≥30 days. Inpatient encounters were pulled from the Merative inpatient services database. Diagnostic claims occurring at any time between the start and end of observed enrollment were eligible for case identification.<br><br>SCD diagnoses were defined using International Classification of Diseases, Ninth and Tenth Revision (ICD-9/10 Codes: 282.41, 282.42, 282.6, D57.00, D57.01, D57.02, D57.211, D57.212, D57.219, D57.411, D57.412, D57.419, D57.811, D57.812, D57.819, D57.1, D57.20, D57.40, D57.80) , encompassing both sickle cell anemia and related genotypes. This case identification approach has been previously validated [1-3] and widely applied in administrative claims research to accurately capture individuals with SCD.<br><br>References:<br>1. Newman TV, Yang JY, Suh K, Jonassaint CR, Kane-Gill SL, Novelli EM. Use of Disease-Modifying Treatments in Patients With Sickle Cell Disease. Jama Netw Open. 2023;6(11).<br>2. Grosse SD, Green NS, Reeves SL. Administrative data identify sickle cell disease: A critical review of approaches in US health services research. Pediatr Blood Cancer. 2020;67(12).<br>3. Reeves SL, Madden B, Wu M, et al. Performance of ICD-10-CM diagnosis codes for identifying children with Sickle Cell Anemia. Health Serv Res. 2020;55(2):310-317.
Outpatient Prescriptions	Outpatient prescription claims were identified using the generic name variable (GENNME) in the MarketScan databases. Opioid medications included morphine, methadone, oxycodone, hydrocodone, hydromorphone, and tramadol. We extracted data on days' supply, dispensed quantity, medication strength, and morphine milligram equivalents (MME), enabling standardized characterization of opioid dispensing.
Monthly Rate of Outpatient Prescriptions Dispensed (Total Prescriptions per 100 Enrollees)	Monthly outpatient prescription rates were calculated as the total number of prescriptions dispensed in a given month divided by the total person-months of enrollment during that month. To account for changes in the size of the underlying population over time and to facilitate comparison across periods and insurance groups, rates were expressed per 100 enrollees, with corresponding standard errors estimated for each month. This approach reduces bias that may arise when absolute prescription counts are influenced by time-varying population exposure at the aggregate level.[1]<br><br>Because sickle cell disease (SCD) is characterized by age-dependent mortality, crude aggregate rates may be distorted by left truncation, whereby individuals must survive to older ages to be observed in later periods.[2] Survivors at older ages may differ systematically from those who do not survive, often representing a healthier subset of the population, which can lead to underestimation or mischaracterization of disease burden if age structure is ignored.<br><br>To address this, we calculated age-stratified monthly prescription rates based on age at the time of prescription and applied direct age standardization. Age standardization reweights age-specific outcome rates to a common reference population, thereby reducing confounding due to shifts in age distribution that result from differential survival over time. Although this approach does not explicitly model delayed entry at the individual level, it provides a population-level adjustment that improves comparability across time and payer groups and mitigates distortions driven by the overrepresentation of older, healthier survivors.[2]<br><br>For age standardization, we selected 2023 as the reference year. For each month, age-specific prescription rates were multiplied by the corresponding age-group weights derived from the reference population and summed to generate a single age-standardized monthly rate. Rates were reported as total prescriptions per 100 enrollee-months.<br><br>Using this approach, we calculated age-standardized monthly rates of total prescriptions for each opioid. Specifically, for each month, prescriptions were aggregated within age groups, age-specific rates per 100 enrollees were computed, and these rates were weighted and summed across age strata.

© 2026 Xu KY et al. *JAMA Network Open*.

	References:<br><br>1. Xu KY, Newman TV, McGill LS, Novelli EM, Hillery CA, Buss JL, Gong L, Huang R, Dong F, Stwalley D, Salas J, Liu SA, Scherrer JF, Brown TR, Park TW, LaRochelle MR, Grucza RA, Jonassaint CR. Ten-year trends in opioid prescribing and vaso-occlusive crises in sickle cell disease: a population-based national cohort study (2011-2022). Lancet Reg Health Am. 2025 Aug 21;50:101214. .<br>2. DeBaun MR, Ghafuri DL, Rodeghier M, et al. Decreased median survival of adults with sickle cell disease after adjusting for left truncation bias: a pooled analysis. *Blood.* 2019;133(6):615-617.
Prescriptions per Person	We estimated the age-standardized mean number of prescriptions dispensed per person per month using direct standardization. Within each month, prescription counts were first summarized at the individual level and averaged within age groups to obtain age-specific monthly means. These age-specific means were then weighted according to the 2023 age distribution of individuals with sickle cell disease and summed across age strata to produce a single age-standardized estimate. When a given month-age stratum was informed by a single observation, within-stratum variability could not be estimated and was therefore assumed to be zero for purposes of standard error calculation.
Average Days Supplied	We calculated age-standardized monthly estimates of average days supplied per dispensed prescription for each opioid. Within each month, average days supplied was calculated by dividing the total days supplied by the number of prescriptions dispensed. Overlapping prescriptions for the same opioid were adjusted to avoid double counting days of therapy before monthly aggregation. Age-specific monthly estimates were then calculated within age strata, weighted to the 2023 age distribution of individuals with sickle cell disease, and summed across age groups to generate a single age-standardized monthly estimate.
Mean Daily Morphine Milligram Equivalents	Daily morphine milligram equivalents (MME) were calculated for each opioid prescription using standard conversion methods. Daily MME was computed as medication strength per unit multiplied by the number of units dispensed per day and the opioid-specific MME conversion factor. Conversion factors were obtained from publicly available Centers for Medicare & Medicaid Services guidance.<br><br>For each opioid, we estimated age-standardized monthly mean daily MME among individuals dispensed that opioid. Age-specific mean daily MME values were calculated within each month, weighted to the 2023 age distribution of individuals with sickle cell disease, and summed across age groups to generate a single age-standardized monthly estimate. To limit the influence of extreme values or potential data entry errors, daily MME values exceeding 1,440 MME/day were truncated before age standardization.

© 2026 Xu KY et al. *JAMA Network Open*.
