# Supplementary material for: Opioid Dispensing Volume and Per-Patient Intensity in Sickle Cell Disease
Source: JAMA Netw Open. Author manuscript; Available in PMC 2026 Oct 1. (PMC13629608; doi:10.1001/jamanetworkopen.2026.32141)
Supplement: Supplementary file 2 [file NIHMS2208928-supplement-Online_Only_Supplement_2.pdf]

# Data Sharing Statement

Xu. Opioid Dispensing Volume and Per-Patient Intensity in Sickle Cell Disease. *JAMA Netw Open*. Published September 04, 2026. doi:10.1001/jamanetworkopen.2026.32141

**Data**
**Data available:** No

**Additional Information**
**Explanation for why data not available:** Data is proprietary
